# The Association of Liver Enzymes With Acute Cholecystitis Diagnosed Using the Tokyo Criteria in the Eastern Region of Saudi Arabia: A Retrospective Case‐Control Study

**DOI:** 10.1002/hsr2.70707

**Published:** 2025-04-16

**Authors:** Mohammed Y. Alessa, Jawad S. Alnajjar, Mohammed A. Almarzooq, Mohammed A. AlSharit, Sarah Talal AlFares, Ahmed E. Alamin, Zuhair Yousef Aljomeah, Loai S. Albinsaad

**Affiliations:** ^1^ General Surgery King Faisal University Alahsa Saudi Arabia; ^2^ College of Medicine King Faisal University Alahsa Saudi Arabia; ^3^ College of Medicine Imam Abdulrahman Bin Faisal University Khobar Saudi Arabia; ^4^ Alhasa Health Cluster Alahsa Saudi Arabia; ^5^ College of Medicine West Kordufan University Sudan

**Keywords:** acute cholecystitis, diagnostic criteria, liver enzyme, sensitivity, Tokyo criteria

## Abstract

**Background:**

Acute cholecystitis, an inflammation of the gallbladder, is often triggered by gallstones but can also result from ischemia, motility issues, chemical harm, and microbial infections. This condition can present with fever, nausea, right upper quadrant pain, and tenderness, influenced by various risk factors including age, gender, genetics, obesity, diet, and lifestyle.

**Aim:**

This retrospective case‐control study investigates the relationship between liver enzymes and acute cholecystitis in patients diagnosed based on Tokyo criteria in Alahsa, Saudi Arabia, from April 2016 to December 2023.

**Methods:**

The study included 504 participants, with 249 cases of acute cholecystitis and 255 controls without cholecystitis, collected from King Fahad Hospital's medical records. Inclusion criteria were patients above 18 years old diagnosed with acute cholecystitis and availability of liver enzyme data. Data were analysed using SPSS version 21, employing descriptive analysis, Pearson Chi‐Square test, Kruskal‐Wallis test, box‐plot visualization, Spearman correlation analysis, and ROC curve analysis.

**Results:**

The mean age was similar between cases (39.9 ± 15.3 years) and controls (40.1 ± 13.9 years). Significant differences were found in gender distribution and BMI. ALT and ALP levels were significantly higher in cases compared to controls (*p* = 0.002 and *p* = 0.001, respectively). The WBC count was also significantly higher in cases (*p* = 0.001). ROC curve analysis indicated that ALT and ALP had good discriminant ability to predict acute cholecystitis. Multiple hierarchical logistic regression showed that higher levels of ALT and ALP were significant predictors for acute cholecystitis, with adjusted odds ratios of 1.21 and 1.24, respectively.

**Conclusion:**

Elevated ALT and ALP levels are significant predictors of acute cholecystitis, demonstrating the importance of liver enzymes in the diagnosis and management of this condition. The findings suggest that integrating liver enzyme measurements with Tokyo criteria can enhance diagnostic accuracy and improve healthcare outcomes for patients with acute cholecystitis.

## 1 Introduction

Acute cholecystitis (AC), an inflammation of the gallbladder, is often triggered by gallstones. However, other factors such as ischemia, motility issues, direct chemical harm, and microbial infections can also play a significant role [1]. Acute cholecystitis can be either calculous, associated with gallstones, or acalculous which is not associated with gallstones [2]. Furthermore, Acute cholecystitis typically presents with symptoms such as fever, nausea, and right upper quadrant pain, that may associated with eating as well as tenderness in the same area on physical examination [3]. In addition to these symptoms, there are several risk factors contribute to the development of cholecystitis and they are unmodifiable risk factors such as female gender, increasing Age, genetic factors, ethnicity, and family history, as well as modifiable risk factors such as Obesity, rapid weight loss, hypertriglyceridemia, drugs lowering‐cholesterol, slow intestinal transit, gallbladder stasis, high calorie diet, highly absorbable sugars, low fiber diet, low calcium, low vitamin C diet, alcohol abstinence, smoking, and sedentary behavior.

The global incidence of AC is estimated to range between 6300 and 20,900 cases per 100,000 individuals this estimation applies to both individuals below the age of 50 years and above the age of 50 years world wide, respectively [3, 4]. Moreover, gallstones‐related pathological conditions, are more prevalent in areas where fast food consumption is common and physical activity levels are low. Meanwhile, In industrialized nations, Cholecystolithiasis is highly prevalent reaching up to 21% on the other hand, In underdeveloped nations, its prevalence is much lower, dropping to as low as 4.1%. Unfortunately, between 1988 and 1994 and 2017 ‐ March 2020, the prevalence of gallstone disease in the US climbed from 7.4% to 13.9%, and the frequency of gallbladder surgery increased from 6.0% to 11.6% [5].

Gallstone disease is becoming increasingly common in Saudi Arabia, which is a reason for concern [6]. It has been observed that in Saudi Arabia, the incidence of AC was 4.4/per 100,000 persons annually, with a considerable prevalence of around 24% and a female‐to‐male ratio of 11.9:1. Our location is situated in one of the highest altitude zones in the Middle East, exceeding 3000 m above sea level [7]. This high altitude might be one of the etiological factors contributing to the high incidence of gallstones in our area [7]. Considering that 50%–85% of sickle cell anemia patients develop pigmented gallstones [8]. The population of Al‐Ahsa, a vast oasis in the eastern region of Saudi Arabia, would be at the highest risk for gallstones in the country [6].

Cholecystitis is optimally treated surgically, but it can also be treated conservatively if needed [2]. Each year, acute cholecystitis results in approximately 700,000 cholecystectomy procedures in the United States, costing around 6.5 billion dollars annually [9]. However, there is a risk of developing complicated cholecystitis if the inflammation persists, potentially leading to gallbladder perforation or gangrene. Fortunately, the mortality rate of Cholecystitis is relatively low, estimated as 0.6%. In terms of diagnosis, acute cholecystitis is identified on the basis of clinical features and is confirmed by ultrasonographic scanning. Without a doubt, Liver enzymes play a crucial role in diagnosing or rule out cholecystitis. A previous study, found that liver enzyme tests, including aspartate aminotransferase (AST), alanine transaminase (ALT), bilirubin, or alkaline phosphatase (ALP), alone or in combination, show variability in patients with acute cholecystitis and/or choledocholithiasis. Interestingly, Ten years have passed since the publication of the Tokyo Guidelines for the diagnosis of acute cholecystitis. Our hypothesis is that using liver's enzymes with Tokyo criteria would increase the accuracy of diagnoses which can lead to better healthcare outcome. Therefore, the aim of our study is to identify the relationships between liver enzymes and the information of patients diagnosed with AC based on Tokyo criteria guidelines.

## 2 Methodology

### 2.1 Study Design

This is a retrospective case‐control study designed to investigate the relationship of liver enzymes in patients diagnosed with acute cholecystitis based on Tokyo criteria in Alahsa, Saudi Arabia, from 2021 to 2022. The inclusion criteria include all male and female patients in King Fahad Hospital above 18 years old diagnosed with acute cholecystitis based on Tokyo criteria and liver enzyme availability from medical records. The exclusion criteria are patients outside King Fahad Hospital, non‐Saudi resident patients, and those less than 18 years old. The study population consists of 504 participants, including 249 patients diagnosed with acute cholecystitis and a control group of 255 individuals without cholecystitis. The data were collected from the medical records of King Fahad Hospital in Al Ahsa, Saudi Arabia, covering the period from April 2016 to December 2023.

### 2.2 Data Collection

The following information were extracted from the medical records of King Fahad Hospital to an outside sheet, sex, age, BMI, nationality, aspartate aminotransferase (AST), alanine transaminase (ALT), alkaline phosphatase (ALP), total bilirubin (T.BILL), D.BILL, and WBC, ultrasonographic findings (US), Computed tomography (CT) findings, Magnetic resonance cholangiopancreatography (MRCP).

### 2.3 Diagnostic Criteria

1. Patient's data, history, and physical examination,
2. Laboratory test: Complete blood count, Liver function test.
3. Imaging: Ultrasonographic findings, CT findings, MRCP findings.

### 2.4 Ethical Considerations

Following the ethical rules for research involving human people, the data were kept private and used only for study. This study was approved by Ahsa Health Cluster's Ethics Committee (41‐EP‐2022).

### 2.5 Data Analysis

The data were collected, reviewed and then fed to Statistical Package for Social Sciences version 21 (SPSS: An IBM Company). All statistical methods used were two tailed with alpha level of 0.05 considering significance if P value less than or equal to 0.05. Descriptive analysis was done by prescribing frequency distribution and percentage for patients' bio‐demographic data and BMI. For liver enzymes, mean with standard deviation, and median level were assessed. Median was calculated due to the skewed data distribution. All parameters were compared between study groups (cases vs. controls) using Pearson Chi‐Square test for categorical variables and Kruskal‐Wallis test for quantitative variables. Box‐plot was used to display WBCs and ALP and ALT by study groups. Spearman correlation analysis was used to assess nature and strength of relation between liver enzymes and assessed patients' WBCs level in both groups. ROC curve analysis with area under curve besides multiple hierarchical logistic regression model based on adjusted odds ratio for patients' age, gender and BMI were used to assess discriminant and predictive ability of liver enzymes for acute cholecystitis.

## 3 Results

A total of 249 cases (acute cholecystitis) and 255 controls were included. The mean age of cases was 39.9 ± 15.3 years old versus 40.1 ± 13.9 years old for controls with no statistical significance (*p* = 0.480). As for gender, 157 (63.1%) of cases were females compared to 186 (72.9%) of controls with statistically significant difference (*p* = 0.017). Considering BMI, 178 (71.5%) of cases were obese compared to 132 (51.8%) of controls while overweight was 17.7% versus 26.7% of controls with mean BMI was 30.6 ± 7.3 for cases and 29.7 ± 6.6 for controls (*p* = 0.001) (Table 1).

**Table 1:** Personal characteristics of study groups (cases and controls), Eastern region, Saudi Arabia.

Personal data	Group				*p* value
	Case (*n* = 249)		Control (*n* = 255)		
	No	%	No	%	
**Age in years**					**0.480**
**< 30**	**70**	**28.1%**	**58**	**22.7%**	
**30–39**	**73**	**29.3%**	**83**	**32.5%**	
**40–49**	**48**	**19.3%**	**57**	**22.4%**	
**50**+	**58**	**23.3%**	**57**	**22.4%**	
**Mean ± SD**	**39.9** ± **15.3**		**40.1** ± **13.9**		
**Gender**					**0.017** *
**Male**	**92**	**36.9%**	**69**	**27.1%**	
**Female**	**157**	**63.1%**	**186**	**72.9%**	
**Body mass index**					**0.001** *
**Normal weight**	**27**	**10.8%**	**55**	**21.6%**	
**Overweight**	**44**	**17.7%**	**68**	**26.7%**	
**Obese**	**178**	**71.5%**	**132**	**51.8%**	
**Mean ± SD**	**30.6** ± **7.3**		**29.7** ± **6.6**		

Abbreviations: P, pearson *X* ^2^ test; ^, exact probability test.

* *p* < 0.05 (significant).

**Figure 1:**
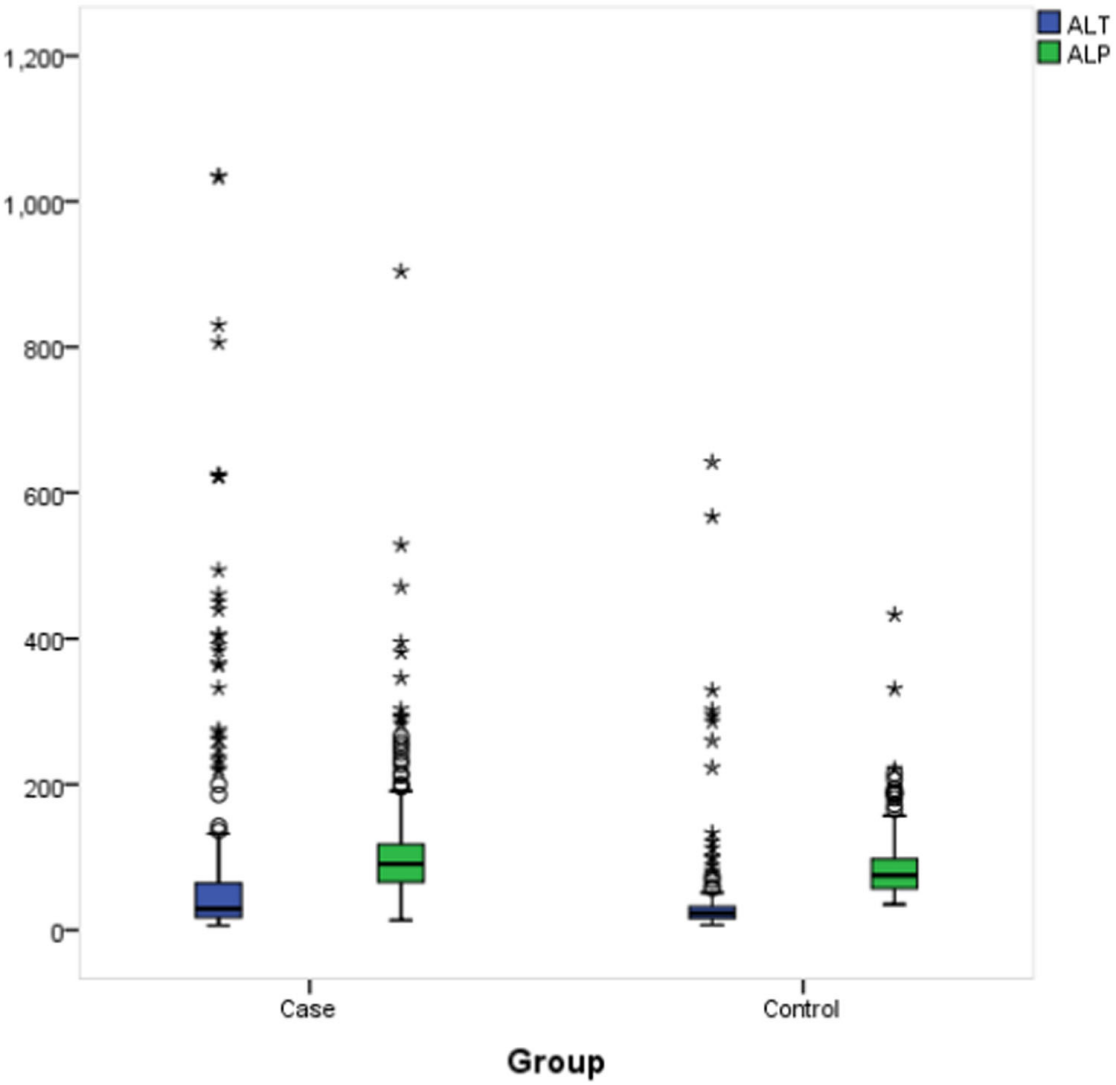
Box‐plot for Liver enzymes profile and descriptive among study groups.

Table 2 and Figure 1. Liver enzymes profile and descriptive among study groups. AST median level was 22.5 among cases versus 24 among controls with no statistical significance (*p* = 0.517). Only ALT was significantly higher among cases than among controls (29 vs. 23, respectively; *p* = 0.002) and also ALP (86.4 vs. 76.0, respectively; *p* = 0.001). Total bilirubin was insignificantly higher among cases (12.1 vs. 10.9) but direct bilirubin was insignificantly higher among controls (4.0 vs. 3.5).

**Table 2:** Liver enzymes profile and descriptive among study groups.

Liver enzymes	Group						*p* value
	Case			Control			
	Mean	SD	Median	Mean	SD	Median	
AST	80.46	144.63	22.50	33.15	33.06	24.00	0.517
ALT	96.53	176.70	29.00	42.48	77.46	23.00	0.002*
ALP	117.60	105.35	89.00	86.38	47.68	76.00	0.001*
Total bilirubin	23.36	42.42	12.10	17.30	19.29	10.95	0.565
Direct bilirubin	14.09	25.19	3.50	7.22	10.54	4.00	0.477

*Note:* P: Mann‐Whitney test.

* *p* < 0.05 (significant).

**Figure 2:**
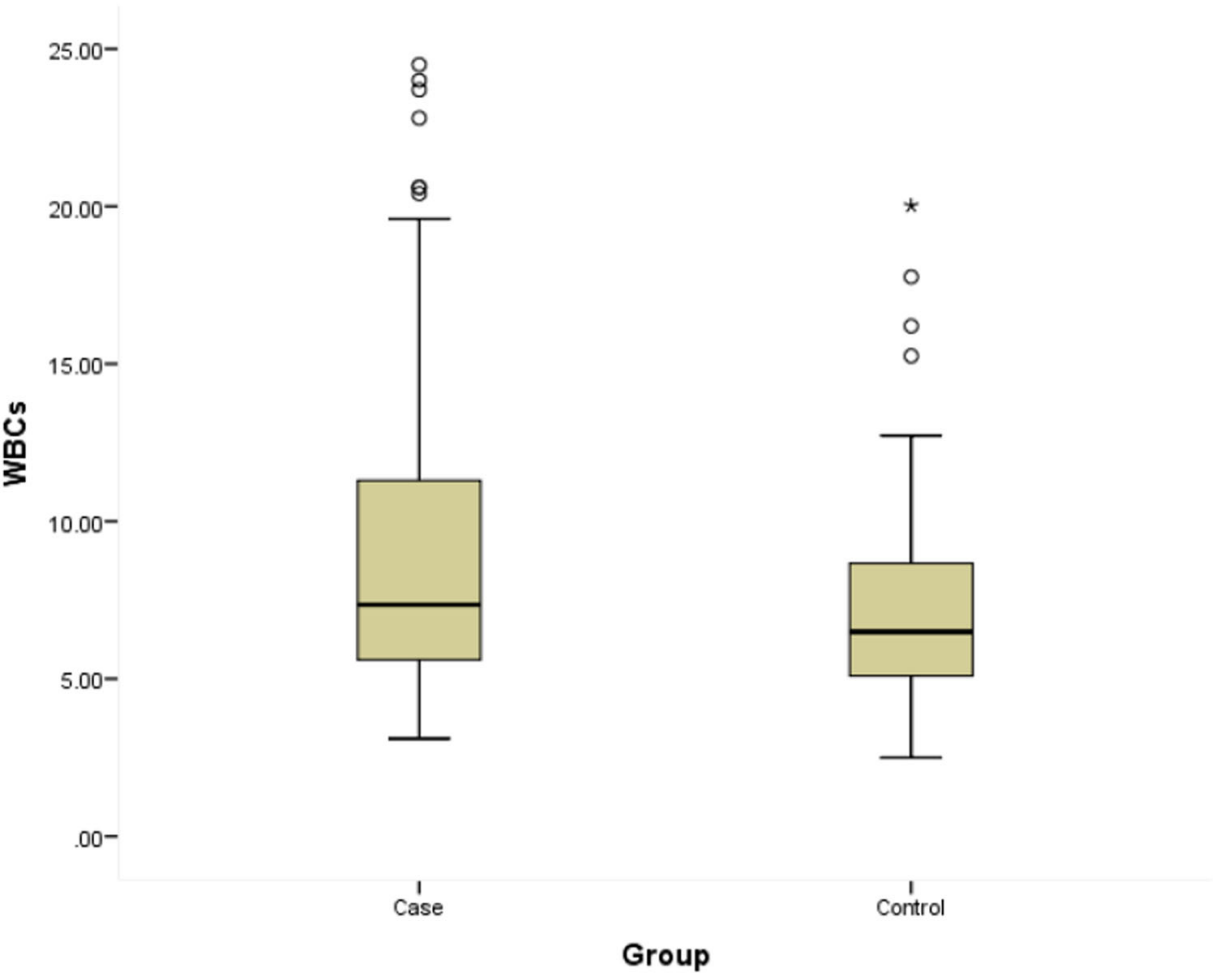
WBCs among study groups (cases with acute cholecystitis vs. controls).

Figure 2 WBCs among study groups (cases with acute cholecystitis *vs.* controls). The mean WBCs level among cases was 9.3 5.1 × 103 versus 7.1 2.6 × 103 among controls with a recorded statistical significance (*p* = 0.001).

Table 3 correlation between liver enzymes and WBCs among study groups. Among cases, only ALT showed a significant weak positive correlation with WBCs count among study patients (rho = −0.18; *p* = 0.049). Among controls, also ALT showed a significant weak positive correlation with WBCs count among study patients (rho = −0.19; *p* = 0.007).

**Table 3:** Correlation between liver enzymes and WBCs among study groups.

Group	Liver enzymes	WBCs	
		rho	*p* value
Case	AST	0.06	0.488
	ALT	0.18	0.049*
	ALP	0.02	0.852
	Total bilirubin	0.11	0.235
	Direct bilirubin	0.03	0.786
Control	AST	0.107	0.121
	ALT	0.197	0.007*
	ALP	−0.094	0.186
	Total bilirubin	0.090	0.168
	Direct bilirubin	0.088	0.366

*Note:* rho: Spearman correlation coefficient.

* *p* < 0.05 (significant).

**Figure 3:**
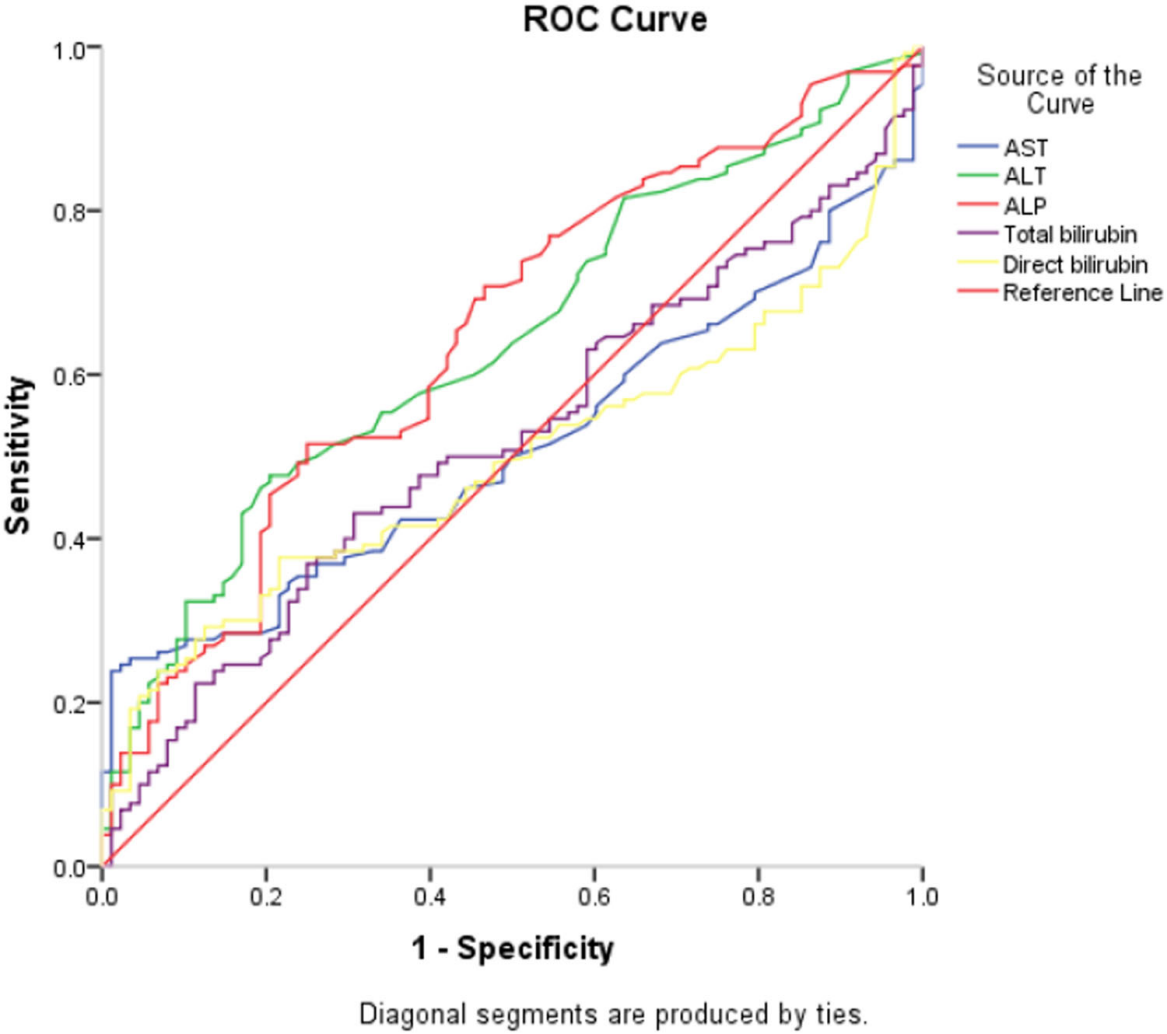
ROC curve for liver enzymes as indicators of acute cholecystitis among study participants.

Table 4 and Figure 3 ROC curve and discriminant ability for liver enzymes as indicators of acute cholecystitis among study participants. Only ALT and ALP showed significant discriminant ability to predict acute cholecystitis with good area under curve (0.64 and 0.66, respectively; *p* = 0.001).

**Table 4:** Discriminant ability (sensitivity) of liver enzymes as predictors for acute cholecystitis.

Liver enzymes	AUC	*p* value	95% CI	
			LL	UL
AST	0.51	0.796	0.434	0.586
ALT	0.64	0.001*	0.567	0.713
ALP	0.66	0.001*	0.574	0.722
Total bilirubin	0.53	0.609	0.444	0.597
Direct bilirubin	0.49	0.950	0.421	0.574

Abbreviations: AUC, area under curve; CI, confidence interval; LL, lower limit; UL, upper limit.

* *p* < 0.05 (significant).

Table 5 multiple hierarchical logistic regression for liver enzymes predictors of acute cholecystitis. After adjustment for patients' age, gender and BMI, only ALT and ALP were the significant predictors for acute cholecystitis where higher ALT levels were associated with 21% more likelihood of acute cholecystitis (AOR = 1.21; 95% CI: 1.01–1.98) and higher level of ALP was associated with 24% more likelihood for acute cholecystitis (AOR = 1.24; 95% CI: 1.11–1.99).

**Table 5:** Multiple hierarchical logistic regression for liver enzymes predictors of acute cholecystitis.

Liver enzymes	*p*‐value	AOR	95% CI	
			Lower	Upper
AST	0.285	1.01	0.99	1.02
ALT	0.048*	1.21	1.01	1.98
ALP	0.047*	1.24	1.11	1.99
T.BILL	0.424	0.991	0.96	1.03
D.BILL	0.901	1.01	0.96	1.04

Abbreviations: AOR, odds ratio adjusted for age, gender, and BMI; CI, confidence interval.

* *p* < 0.05 (significant).

## 4 Discussion

The utilization of Tokyo guideline criteria in diagnosing and severity grading of Acute cholecystitis is a recommendation 1, level C by the last Tokyo consensus in 2018 [10]. Moreover, the application of the Tokyo guideline criteria is widely used worldwide. Hudgi A. et al. did a comparison assessment between the application of the Tokyo guideline and the fellow assessment. It revealed that the diagnostic accuracy of the Tokyo guideline criteria is 81% and it reduces the false positive rates [11]. However, the accuracy as per the consensus reached up to 83.1% [10]. In addition, the accuracy of Tokyo guideline criteria suggests the usefulness of this tool in the diagnostic process of patients with acute cholecystitis.

In attempts to improve the accuracy of the Tokyo guideline criteria there were multiple studies suggested the use of other diagnostic tools for example, Liver function test. Talukdar M. et al. study showed that liver enzymes and bilirubin tends to transiently increase in cases of acute cholecystitis [12]. In addition, Zgheib H. et al. suggested that an abnormal liver function is a predicator for the presence of Common Bile duct (CBD) stones with the presence of acute cholecystitis [13]. However, Song S. H. et al. study showed elevated liver enzymes in patients with acute cholecystitis may not indicate the presence of cholelithiasis whereas it might indicate fatty liver disease [14]. In summary, multiple studies showed the presence of elevated liver enzymes in cases of acute cholecystitis with the possibility of the presence of other pathological entities that is, the use of liver function might aid in the diagnostic process of such patients.

In this study, ALT and ALP showed a statistically significant increase in cases with acute cholecystitis compared to the control group, suggesting that ALT and ALP are strong predictors for acute cholecystitis. In addition, ALT and ALP were previously observed in the literature to be present in cases of acute cholecystitis with a percentage of 49% and 32% respectively [12]. Furthermore, females showed the majority of the cases with 63.1%. Also, the majority of the cases with a percentage of 71.5% who is classified as obese according to the body mass index (BMI) showed a statistically significant difference between the two groups. However, the association between obesity, ALT, and ALP was assessed and it revealed that there is no associated between the two [15]. Finally, The utilization of liver enzyme test specifically (ALT and ALP) adjacently with the Tokyo criteria guidelines shows a promising results in the diagnostic process of acute cholecystitis cases.

## 5 Limitations

This study has several limitations. As a retrospective case‐control study, it relies on existing medical records, which may be incomplete or inaccurate, particularly regarding liver enzyme data and patient demographics. Conducted at a single hospital, King Fahad Hospital in Al Ahsa, Saudi Arabia, the findings may not be generalizable to other populations. Selection bias is possible, as only patients with available liver enzyme data were included, potentially excluding some acute cholecystitis cases. Furthermore, other hepatic or biliary conditions that could also elevate ALT and ALP levels were not systematically excluded, potentially limiting the diagnostic utility of these enzymes. The study's cross‐sectional nature limits the ability to establish causality between elevated liver enzymes and acute cholecystitis. Additionally, not all potential confounders, such as comorbid conditions and lifestyle factors, were accounted for. The study period from 2021 to 2022 may not capture long‐term trends or seasonal variations. Future research with larger, more diverse populations and prospective designs is needed to validate these findings and explore underlying mechanisms.

## 6 Conclusion

This retrospective case‐control study underscores the importance of liver enzymes, specifically ALT and ALP, in diagnosing acute cholecystitis using Tokyo criteria. Elevated levels of these enzymes were significant predictors of the condition. The study also highlighted significant differences in gender distribution and BMI between cases and controls, emphasizing the multifactorial nature of acute cholecystitis. The correlation between liver enzymes and WBC counts suggests that inflammatory markers are crucial in the diagnostic process. These findings support the integration of liver enzyme measurements with Tokyo criteria guidelines to improve diagnostic accuracy and healthcare outcomes for patients with acute cholecystitis.

## Author Contributions

**Mohammed Y. Alessa:** conceptualization, supervision, writing – review and editing, resources. **Jawad S. Alnajjar:** data curation, writing – review and editing, writing – original draft, conceptualization. **Mohammed A. Almarzooq:** formal analysis, writing – original draft, software. **Mohammed A. AlSharit:** writing – review and editing, formal analysis, visualization. **Sarah Talal AlFares:** writing – review and editing, data curation, investigation. **Ahmed E. Alamin:** methodology, software, writing – original draft. **Zuhair Yousef Aljomeah:** data curation, software, writing – review and editing. **Loai S. Albinsaad:** funding acquisition, validation, project administration.

## Conflicts of Interest

The authors declare no conflicts of interest.

## Transparency Statement

The lead author Jawad S. Alnajjar affirms that this manuscript is an honest, accurate, and transparent account of the study being reported; that no important aspects of the study have been omitted; and that any discrepancies from the study as planned (and, if relevant, registered) have been explained.

## Data Availability

All relevant data supporting the findings of this study are included within the manuscript.
